# Symbiotic 7: Integration of Predator and More (Competition Contribution)

**DOI:** 10.1007/978-3-030-45237-7_31

**Published:** 2020-03-13

**Authors:** Marek Chalupa, Tomáš Jašek, Lukáš Tomovič, Martin Hruška, Veronika Šoková, Paulína Ayaziová, Jan Strejček, Tomáš Vojnar

**Affiliations:** 8 Johannes Kepler University, Linz, Austria; 9 University of Birmingham, Birmingham, UK; 10 Masaryk University, Brno, Czech Republic; 11 Brno University of Technology, FIT, IT4Innovations Centre of Excellence, CZ Brno, Czech Republic

## Abstract

Symbiotic  7 brings improvements in all parts of the tool. In particular, we integrated the advanced shape analysis implemented in Predator to our instrumentation process for memory safety checking. Further, we extended our slicer to correctly handle non-terminating programs. This new slicing is applied in termination analysis, where we also added instrumentation for detection of simple cycles in the program state space. The witness generation process changed as well.

## 1 Verification Approach

Symbiotic 7 follows the same basic schema as all previous versions [4,5]: the program to be verified is first instrumented (if needed), then reduced by static program slicing, and finally symbolically executed using Klee [2]. We describe the main modifications since Symbiotic 5 (participating in SV-COMP 2018) as modifications in Symbiotic 6 (competing in 2019) have not been published.

**Memory safety checking improvements** Symbiotic uses a static pointer analysis to detect instructions that can potentially violate memory safety. To check these instructions, Symbiotic 5 [5,3] instrumented the program with code that keeps records about allocated memory and uses the records to assert the validity of potentially misbehaving instructions. Then we sliced the program with respect to these assertions and called Klee to check assertion validity.

Since Symbiotic 6, we slice the program directly with respect to the potentially misbehaving instructions without inserting any additional code. Then we call Klee to check memory safety of the sliced program.

Symbiotic 7 newly integrates Predator [6], a static analyzer specialized on memory safety. We first run Predator in its over-approximating mode and

* M. Chalupa, T. Jašek, P. Ayaziová, and J. Strejček have been supported by the Czech Science Foundation grant GA18-02177S. M. Hruška, V. Šoková, and T. Vojnar have been supported by the IT4Innovations Excellence in Science project (LQ1602) and the FIT BUT internal project FIT-S-20-6427.

** Jury member and corresponding author: chalupa@fi.muni.cz.

in a configuration that analyses all branches in the given program and tries to recover from found errors. If PREDATOR says that the program is safe, we simply answer *true*. Otherwise, we take bug reports from PREDATOR and combine them with results of our static pointer analysis to get a more precise (i.e., smaller) set of potentially misbehaving instructions. Then we proceed like SYMBIOTIC 6.

SYMBIOTIC 7 is also the first version that can distinguish between *valid-memcleanup* and *valid-memtrack* properties. To do this, our clone of KLEE now reconstructs the shape of memory at the program exit if unfreed memory is found: KLEE starts with local and global variables and resolves pointers in these (if any). Then it resolves pointers in the pointed memory, etc. This way we can find out if the unfreed memory is reachable via a chain of dereferences or not.

**Termination analysis** SYMBIOTIC 6 introduced a simple support for termination property: a call to `__VERIFIER_error` is inserted before trivial infinite loops, e.g., `while (true);` loops. If the symbolic execution detects that such a call is reachable, SYMBIOTIC answers *false* as the program can reach an infinite loop. If all paths of the program are explored by symbolic execution without reaching any of these calls, all program executions are clearly terminating and we answer *true* (an infinite program path cannot be fully explored by symbolic execution). Note that program slicing was disabled for non-termination checking in SYMBIOTIC 6 as the slicer could remove infinite loops in some specific cases.

SYMBIOTIC 7 brings two improvements. First, since we extended our slicer to correctly handle non-terminating programs [7], we now apply slicing with slicing criteria set to all exit points (including the instrumented error calls) of the program. Second, we instrument the program with checks for simple cycles in the state space. The instrumentation detects non-nested loops with a single entry for which it can conservatively determine a set $\{V_1, \ldots, V_k\}$ that includes all variables potentially modified by the loop. At the beginning of the loop body, we insert assignments that store the value of each variable $V_i$ into a new variable $V_i'$. At the end of the loop body, we insert the assertion $\texttt{assert}(V_1 \neq V_1' \vee \ldots \vee V_k \neq V_k')$ to check a change in the vector of these variables. If this assertion is violated, the program has a non-terminating execution.

**Error path replay** Although the slicer in SYMBIOTIC now provides algorithms that preserve non-termination properties of programs, outside the *Termination* category we still use the original *non-termination insensitive* slicing as it may remove more instructions. The price is, however, that SYMBIOTIC may report false alarms: an unreachable error location situated below an infinite loop may become reachable when the loop is sliced out. To fix this issue, we try to reproduce each error found by symbolic execution in the original (unsliced) program. If the error is reproduced, we report it as a real error. Otherwise, we say *unknown*.

**Improved witness generation** SYMBIOTIC 5 and 6 generated violation witnesses that describe only the initialization of non-deterministic variables at the beginning of the `main` function. Symbiotic 7, on the other hand, generates violation witnesses that contain a complete test vector, i.e., the whole sequence of values returned from `__VERIFIER_nondet_*` functions during the error path replay. To get and correctly identify all these values, we have modified our fork of Klee to support interpretation of `__VERIFIER_nondet_*` functions (and other undefined functions in general) internally. Currently, more than 99% of our violation witnesses (outside the *Termination* category) are confirmed. Symbiotic 7 still generates trivial correctness witnesses if no error is found.

**Other improvements** Other improvements in Symbiotic 7 used in SV-COMP 2020 include a faster data dependence analysis (a part of slicing) and better handling of `assume` statements in the slicer. Symbiotic is now also able to continue in verification if the instrumentation or slicer crashes or exceeds the time limit. In such a case, Klee is run on the original program which has been only optimized by standard llvm optimizations. For SV-COMP 2020, we set the time limit of 400 s on instrumentation and the time limit of 300 s on slicing.

## 2 Software Architecture

Symbiotic 7 is built on top of llvm 8.0.1 [8]. The tool consists of a set of modules written in C++ that process llvm bitcode, and Python scripts that chain these modules according to given configuration.

For use in Symbiotic, we have made several bugfixes in Predator's llvm backend and ported it to llvm 8.0.1. Further, we have introduced distinguishing between safe and possibly erroneous program instructions.

Symbiotic uses its own fork of Klee that contains several modifications compared to the mainstream Klee. In particular, the fork has been extended to handle symbolic-sized memory allocations, to process marks delimiting the lifetime of scoped variables, to check for memory leaks, and to generate violation witnesses in the SV-COMP format.

## 3 Strengths and Weaknesses

In SV-COMP 2020 [1], Symbiotic 7 won the *SoftwareSystems* category and scored second in the *MemSafety* category and the *FalsificationOverall* meta category. Overall, Symbiotic ended up on the fourth place.

The main reason for winning *SoftwareSystems* is having only a few incorrect answers. Indeed, Symbiotic did not win in the number of correct answers in any of the *SoftwareSystems* subcategories. However, we had only 4 incorrect answers and all of them in the subcategory *DeviceDriversLinux64*. This subcategory is huge and these incorrect answers have only a small impact on the weighted score.

In *MemSafety*, we took the second place after PredatorHP which executes several instances of the Predator tool with different configurations in parallel. Symbiotic calls just one of these instances as mentioned above. Additionally,

PredatorHP uses gcc, while we use Predator running on llvm, which is not as mature as the former. Also, we had a number of new *unknown* answers because Klee does not support pointer comparisons, which we incorrectly did not detect in the previous versions of Symbiotic.

In general, Symbiotic's results stems from the good performance of Klee supported by efficient static analysis and slicing: the official results show that Symbiotic can decide many benchmarks very quickly.

The main weakness of our tool is the inherent complexity of symbolic execution and the limited possibility of analysing potentially unbounded loops or infinite paths with this technique. Indeed, as symbolic execution actually follows all paths in the program, it does not terminate if the program contains an unbounded loop or an infinite path (unless an error is found). Even when the number of paths is finite and all the paths are finite, symbolic execution usually runs out of resources if the number of paths is large. Although this problem is slightly alleviated by program slicing, our tool still does not scale well on complex programs.

## 4 Tool Setup and Configuration

– *Download:* From the competition archives or via http://doi.org/10.5281/zenodo.3678328.
– *Installation:* Unpack the archive.
– *Participation Statement:* Symbiotic 7 participates in all categories.
– *Execution:* Run `bin/symbiotic --sv-comp OPTS <source>`, where available `OPTS` include:
  • `--prp=file`, which sets the property specification file to use,
  • `--witness=file`, which sets the output file for the witness,
  • `--32`, which sets the 32-bit environment,
  • `--help`, which shows the full list of possible options.

## 5 Software Project and Contributors

Symbiotic 6 and 7 have been developed by M. Chalupa, T. Jašek, M. Vitovská, M. Šimáček, L. Tomovič, and P. Ayaziová under the supervision of J. Strejček. Predator has been adjusted for the described integration by M. Hruška and V. Šoková under the supervision of T. Vojnar. Symbiotic and its components are available under the MIT license. The project is hosted by the Faculty of Informatics, Masaryk University. Klee, llvm, and Predator are also available under open-source licenses. Source codes of the project and references to all its components can be found at:

https://github.com/staticafi/symbiotic
